# Human and Veterinary Medicine Collaboration: Synergistic Approach to Address Antimicrobial Resistance Through the Lens of Planetary Health

**DOI:** 10.3390/antibiotics14010038

**Published:** 2025-01-06

**Authors:** Olga Horvat, Zorana Kovačević

**Affiliations:** 1 Department of Pharmacology and Toxicology, Faculty of Medicine, University of Novi Sad, Hajduk Veljkova 3, 21000 Novi Sad, Serbia; olga.horvat@mf.uns.ac.rs; 2 Department of Veterinary Medicine, Faculty of Agriculture, University of Novi Sad, Trg Dositeja Obradovica 8, 21000 Novi Sad, Serbia

**Keywords:** antimicrobial resistance, antimicrobial use, one health, planetary health

## Abstract

Antimicrobial resistance (AMR) represents a critical threat to human, animal, and environmental health, challenging global efforts to maintain sustainable ecosystems and public health systems. In this review, the complex, cross-disciplinary issues of AMR are explored within the framework of planetary health, emphasizing the interconnectedness of human and veterinary medicine with broader environmental and social systems. Specifically, it addresses the social, economic, environmental, and health dimensions of AMR under the planetary health framework. The social aspects consider how public awareness, education, and healthcare practices shape antimicrobial use (AMU) and resistance patterns. The economic impact evaluates the cost burdens of AMR, including healthcare costs, loss of productivity, and the implications for the livestock and food production industries. The environmental dimension highlights the role of pharmaceutical waste, agricultural runoff, and industrial pollution in contributing to the spread of antimicrobials and resistant pathogens in ecosystems. To illustrate these challenges, a comprehensive literature review using the PubMed and Web of Science databases was conducted, identifying 91 relevant articles on planetary health and AMR. In this review, the knowledge from these studies and additional references is integrated to provide a holistic overview of the AMR crisis. By applying the four pillars of planetary health—social, economic, environmental, and health knowledge—in this manuscript, the necessity is underscored of collaborative strategies across human and veterinary medicine to combat AMR. Ultimately, this synergistic approach aims to shape the policies and practices that safeguard public health, protect ecosystems, and promote a sustainable future by implementing antimicrobial stewardship programs and encouraging prudent AMU.

## 1. Introduction

Antimicrobial resistance (AMR) is one of the most critical global health challenges, causing 670,000 infections and nearly 33,000 deaths each year in the European Union [1]. In a vast number of countries, antibiotics are overused to prevent and treat bacterial infections in humans and animals [2,3]. This overuse includes frontline antibiotics like fluoroquinolones, third- and fourth-generation cephalosporins, and carbapenems, contributing to the rise of multidrug resistance [4]. Approximately 66% of antimicrobials are used in animal production [5], with resistance also widespread there, even on organic farms that limit antimicrobial use (AMU) [6]. The disposal of untreated human and livestock waste containing antimicrobial resistant genes and antibiotic residues into natural environments often leads to the emergence of resistant bacteria. These bacteria can then be transmitted to humans through the food chain, significantly impacting health [7]. The climate crisis is already influencing patterns of infectious diseases including the increase in and spread of many bacterial and vector-borne diseases, which may lead to an increase in AMU and rising AMR in humans, animals, plants, and the environment [8]. The aforementioned, complex problem necessitates a multi-sectoral collaboration and action known as the one health approach. This approach clearly focuses on consequences, responses, and actions at the animal–human–ecosystem interfaces, particularly regarding emerging and endemic zoonoses, as well as AMR [8]. Furthermore, AMR can arise in humans, animals, or the environment, and may spread between them and across countries, impacting food safety [2,4]. Despite its widespread and growing acceptance, the one health approach has faced criticism for overemphasizing emerging zoonotic diseases, insufficiently incorporating environmental concepts and expertise, and lacking attention to social science and behavioral aspects of health and governance [9,10]. Additionally, while the veterinary community has embraced the one health concept, the medical community has been slower to fully engage [11]. Although AMR is a natural phenomenon, the main drivers of its development and spread are “man-made”, such as the misuse and overuse of antimicrobials in humans, animals, and plants [12]. Several researchers have demonstrated a positive correlation between AMR and anthropogenic activities, highlighting the role of environmental compartments (soil, water, and air) in the spread of AMR [13]. An increasing number of studies indicate that the problem of AMR is associated with climate change—one of the greatest threats to our planet’s biosphere [13,14]. These challenges reveal that AMR is linked to human activities in the natural environment, necessitating the inclusion of AMR in the planetary health agenda. Planetary health is an emerging field that explores the connections between human health and the natural environment [15]. The Rockefeller Foundation–Lancet Commission on Planetary Health clearly defined this concept as “the achievement of the highest attainable standard of health, wellbeing, and equity worldwide through judicious attention to the human systems—political, economic, and social—that shape the future of humanity and the Earth’s natural systems that define the safe environmental limits within which humanity can flourish” [16]. Planetary health recognizes that human health and well-being are deeply connected to planetary systems, emphasizing the need to preserve the integrity of the natural environment to secure the long-term health of human populations [17]. In their review, Lerner and Berg (2017) attempted to explain the relationship between one health and planetary health [16], describing one health as a concept that emphasizes the importance of interdisciplinarity, as well as the interconnectedness of public health, animal health, and ecosystem health. In contrast, they view planetary health as having a more ’anthropocentric’ focus, seeing ecosystems—including animals as part of the biotic environment—primarily in terms of their contributions to human health, well-being, and sustainability. Therefore, the aim of this study was to explore the planetary health agenda in the context of AMR, highlighting the interconnectedness between human and veterinary medicine. In this study, the health, economic, environmental, and social aspects of AMR are addressed within the framework of planetary health.

## 2. Health Impact of Antimicrobial Resistance Within the Framework of Planetary Health

The health impact of AMR within the framework of planetary health is profound, affecting human, animal, and environmental health in interconnected ways. Hence, there are several health aspects dedicated to this global health issue, such as human and animal health, as well as environmental health and impact of climate change.

### 2.1. Key Health Aspects of Antimicrobial Resistance

The most immediate and visible consequence of AMR within the human health aspect is the growing inability to treat common infections, leading to prolonged illness, higher mortality rates, and increased healthcare costs [18,19]. This leads to higher rates of illness and death globally, particularly in vulnerable populations, such as young children, the elderly, and those with compromised immune systems [20,21]. Moreover, AMR can lead to prolonged illness and increased complications, as infections become harder to treat. Patients may experience a longer duration of symptoms and more severe disease progression, which in turn increases the burden on healthcare systems.

Furthermore, AMR also threatens medical advances, particularly in areas such as surgery, cancer therapy, and organ transplantation, where antibiotics play a crucial role in preventing infections [22]. Without effective antimicrobials, the risk of infections in these settings rises dramatically, compromising patient outcomes and overall public health. The increasing burden of AMR-related diseases also puts pressure on healthcare systems, particularly in low- and middle-income countries, where access to second- or third-line antibiotics is often limited [23].

AMR affects not only humans but also animals, as resistant infections spread among livestock, poultry, and companion animals [24,25] leading to severe implications for veterinary medicine, as treatment failures in animals can lead to economic losses for the farmers and the food industry. In addition, there are also severe consequences on human health [26]. In fact, in livestock production, the overuse of antibiotics to promote growth and prevent disease has contributed to the emergence of resistant bacteria, which can be transmitted from animals to humans through direct contact or the food chain [27,28].

One more important health aspect is that AMR is closely linked to environmental health, as antibiotic pollution from pharmaceutical manufacturing, agricultural runoff, and improper waste management introduces resistant bacteria into natural ecosystems. These bacteria can persist in soil, water, and air, creating reservoirs of resistance that re-enter human and animal populations [29]. Within the planetary health framework, this environmental dimension is critical, as it underscores the need to protect ecosystems from the harmful effects of antibiotic contamination [30].

Finally, climate change further complicates the health impacts of AMR, as shifting ecosystems and altered disease vectors may lead to the emergence of new resistant infections or the spread of existing ones to new areas. Actually, climate change could affect AMR either directly (by changes in temperature and/or precipitation) or indirectly—such as shifts in human populations, disease vectors, agriculture, water availability, glaciation, and hydrology—affect the development or transfer of AMR in bacteria [31]. Finally, climate change may affect human behavior and other social determinants of infectious disease risk; for example, warmer weather resulting in changed food preparation and consumption patterns, or increasing countryside visits, in turn increasing the potential exposure to infectious disease hazards [32].

The planetary health approach, therefore, emphasizes the need for global cooperation and integrated strategies that address both the ecological and health dimensions of AMR.

### 2.2. Strategies for Mitigating Health Impact of Antimicrobial Resistance

Mitigating the health impact of AMR requires a multifaceted approach, involving coordinated efforts from healthcare systems, agriculture, environmental management, and policy frameworks. Since AMR affects human, animal, and environmental health, effective strategies must be grounded in the principles of one health and planetary health, addressing the interconnected nature of these systems. By promoting responsible AMU, strengthening the healthcare infrastructure, and investing in alternatives and surveillance, societies can mitigate the health consequences of AMR while protecting the future efficacy of antibiotics.

Furthermore, engaging communities and raising public awareness about the importance of responsible AMU is essential for changing behavior and reducing unnecessary prescriptions [33,34]. Educational initiatives that target both healthcare professionals and the general public can empower individuals to make informed choices about AMU and adherence to treatment protocols [35]. Additionally, collaboration among stakeholders, including policymakers, researchers, and industry representatives, is crucial for developing comprehensive policies that integrate AMR mitigation strategies across various sectors.

Investing in research and innovation is also vital for discovering new antimicrobial agents and alternative therapies. While vaccines and bacteriophage treatments offer promising avenues to alleviate reliance on traditional antibiotics, antimicrobial peptides, particularly bacteriocins derived from bacteria, also emerge as a highly promising alternative that warrants attention [36,37]. Moreover, improving global surveillance systems will enhance the understanding of AMR trends and facilitate prompt responses to emerging threats [38].

Ultimately, a unified commitment to tackling AMR through the planetary health approach not only protects public health but also ensures the sustainability of our ecosystems, preserving the effectiveness of antibiotics for generations to come.

## 3. Economic Impact of Antimicrobial Resistance Within the Framework of Planetary Health

In addition to posing a significant threat to global health, AMR has a substantial impact on economies worldwide. There are a lot of estimations where the rising incidence of resistant pathogens threatens the effectiveness of existing treatments, leading to prolonged illnesses, increased mortality, and higher healthcare costs [39,40,41,42]. In addition, reduced productivity is present in both the medical and veterinary sectors due to rising prevalence of AMR in humans and animals [24]. In this section, the economic implications of AMR are explored within the broader context of planetary health, a concept that underscores the interconnectedness of human health, animal health, and the environment.

### 3.1. The Economic Burden of Antimicrobial Resistance

AMR also stands to have a profound impact on the global economy [43]. Moreover, the economic impact of AMR is multifaceted, encompassing direct costs such as healthcare expenditure and indirect costs related to lost productivity and diminished quality of life. Globally, it is estimated that AMR not only perhaps will be responsible for 10 million deaths annually, but it could cost the world up to USD 100 trillion by 2050 if no action is taken, emphasizing the urgent threat of AMR [44]. The World Bank’s 2017 report, Drug-Resistant Infections: A Threat to Our Economic Future, estimated that antimicrobial resistance (AMR) could reduce global GDP by 3.8% by 2050 in a high-impact scenario, with annual losses of USD 3.4 trillion by 2030 [43]. The financial burden is particularly pronounced in low- and middle-income countries where the healthcare infrastructure is often inadequate for effectively managing resistant infections [45]. Furthermore, not only do people not have full access to novel antibiotics, which are often more expensive, but also they often use common antibiotics without prescription in those countries [46]. Consequently, low-income countries would experience larger drops in economic growth than wealthy countries, so economic inequality between countries would increase [43]. On the other hand, it is also evident in high-income countries with advanced medical systems [47].

An increase in healthcare costs due to the treatment of resistant infections typically requires more expensive and prolonged therapies [48]. This includes the use of last-resort antibiotics, prolonged antibiotic treatment, extended hospital stays, and additional diagnostic tests [49,50,51,52]. The cumulative effect significantly inflates healthcare budgets. The majority of economic evaluations on AMR interventions have concentrated on methicillin-resistant *Staphylococcus aureus* (MRSA), highlighting a substantial lack of focus on other priority pathogens. Even in cases where studies exist, they often fall short of providing a thorough economic analysis, despite the fact that essential components like intervention costs, bed–day expenditures, and patient outcomes are generally accessible [48]. One important component is productivity losses due to infections with resistant pathogens which can lead to extended sick leave, reduced labor efficiency, and in severe cases, death [53]. This loss of a productive workforce further strains economic systems, especially in sectors heavily reliant on human labor. A review paper that aimed to assess alternative methods for estimating the burden of AMR concluded that more evidence is needed on the secondary impacts of AMR on health, healthcare systems, and the economy indicating that the different perspectives of AMR financial burden produce varying outcome measures [54]. Obtaining estimates for these different outcomes may require different methodologies, ranging from case-control studies with regression analyses to complex mathematical and economic models [55,56].

Global coordination is critically lacking, with piecemeal containment efforts unable to match the evolutionary capacity of pathogenic bacteria continually exposed to humanity’s extensive AMU across healthcare, agriculture, and the environment [57]. The one health concept, endorsed by the World Health Organization (WHO) and the World Organization for Animal Health (WOAH), recognizes the interconnection between sectors and it is therefore essential for both animal and human health to ensure prudent AMU in food-producing animals [58]. Antimicrobials have historically been commonly used in animal treatment not only for infection control but also as growth promoters and feed efficiency enhancers [59]. For instance, in Europe, the use of antibiotics as growth promoters began declining with regulatory interventions starting in 1972, culminating in a complete ban in 2006 [60], which resulted in a measurable decline in antimicrobial consumption in livestock. According to the European Medicines Agency (EMA), between 2011 and 2020, AMU in food-producing animals decreased by over 43% in Europe, demonstrating the effectiveness of regulatory measures [61]. However, the overuse and misuse of these drugs in animals have contributed significantly to the rise of resistant bacterial strains, which can then spread to humans through direct contact, the environment, or the food chain [62,63,64,65]. To better understand the impact of antimicrobial use (AMU) in animal production on antimicrobial resistance (AMR) in humans, Innes et al. [64] developed a model of AMR dynamics and associated externalities. This model quantifies the costs of AMU in animal agriculture on human AMR, using parameters such as (a) the health and economic burden of AMR in humans, (b) the impact of AMU in animal agriculture on AMR in animals, (c) the proportion of human AMR attributable to animal agriculture, and (d) the extent of AMU in animals. Despite the variability in estimates regarding the economic costs of AMR, the current and future implications of AMR underscore the need for immediate action.

One of the key strategies recommended by WHO [65], WOAH [65], and FAO [66] is the reduction in and potential limitation of antimicrobial use (AMU) in animal agriculture. This approach aims to curb the development of resistance by minimizing unnecessary AMU while promoting sustainable practices within the livestock sector. Furthermore, one of the core principles of antimicrobial stewardship (AMS) in veterinary medicine is prudent AMU, which involves the restriction of medically important antimicrobials for human medicine [67]. However, it is crucial to note that many antibiotics used in veterinary and farming contexts, even if different from those used in human medicine, often belong to the same chemical families, such as cephalosporins or quinolones. This overlap raises similar concerns about bacterial resistance, as these related molecules can exert comparable selective pressures, even if they are not identical.

Moreover, the OIE List of Antimicrobial Agents of Veterinary Importance, which includes specific recommendations on AMU, is also of particular importance for AMS while OIE’s strategy on AMR and prudent AMU is aligned with the global action plan on AMR and recognizes the importance of a one health approach [66]. Finally, several countries have shown that AMS, on the basis of detailed information on AMU, AMR, or both, rather than strict inhibition, might lead to desired reductions [67,68]. The veterinary sector should be given a chance to at least try to reduce AMU in animals rather than be imposed upon to do so [69,70].

AMR also affects the agriculture sector, particularly in livestock production, aquaculture, and crop farming leading to economic losses [45,71]. Actually, resistant infections in livestock can lead to decreased animal productivity, increased veterinary costs, and potential trade restrictions on the animal products [72,73]. This not only impacts farmers but also has broader implications for food security and trade [74,75].

The strong link between AMU and the development and spread of AMR in animals is well documented [61,76], leading to a variety of actions and recommendations aimed at reducing AMU in animals. It is the focus of many research projects the aim of which is to combat AMR on development and implementation of antimicrobial alternatives and AMU guidelines [76,77,78,79]. Special attention is also paid to raising awareness about AMR among not just veterinarians, but also all current and future healthcare professionals [62,80,81]. Additionally, the need for innovative strategies to combat AMR should require investment in both the development of new antibiotics and complementary technologies, such as vaccines [82,83,84,85,86,87].

### 3.2. The Impact of Climate Change on Antimicrobial Resistance Through the Lens of Planetary Health and Economic Costs

Planetary health, a concept that emphasizes the interconnectedness of human health, animal health, and ecosystem integrity, highlights the need for addressing climate change as a key driver of global health challenges, including AMR. Climate change and disease risks, including AMR, are two of the most pressing challenges of the Anthropocene and cannot be considered in isolation [88]. Only a few authors have evidenced the links between AMR and climate change explicitly [14,89,90], synthesizing evidence of shared processes exacerbating both threats [91]. Furthermore, examples linking AMR and the climate crisis examine how climate change affects concentrations of heavy metals and biocides in soil and water, which can accelerate bacterial growth and facilitate horizontal gene transfer of resistance. Climate change influences AMR through multiple pathways, including rising temperatures, flooding, and the subsequent increases in population density and displacement also contribute to a higher incidence of waterborne infections [92]. For instance, rising temperatures have been linked to an increased prevalence of resistant pathogens in water systems and soils, posing a risk for zoonotic transmission and foodborne infections [93,94,95]. This, in turn, puts added strain on healthcare systems and water, sanitation, and hygiene (WASH) infrastructures, raising the risk of infections caused by antibiotic-resistant pathogens [92]. Another example of this link is greenhouse gas emissions which were described to be already driving deintensification in some profitable sectors within some countries with intensive high-stocking agriculture. Livestock, such as cows, emit methane and intensive farming frequently involves excessive antibiotic use, contributing to AMR [96]. In turn, a growing consideration of the environmental costs of current actions was said to continue to impact how food is produced, such as a greater orientation to sustainable practices that could have implications for AMU and efforts to prevent AMR development and spread [97]. As a consequence, food safety and security are major concerns in the context of climate change and AMR [91]. Moreover, the economic consequences of climate-driven AMR are significant. As infections become harder to treat, the demand for more effective antibiotics—some of which may be more expensive—continues to grow, leading to increased healthcare costs. Furthermore, the livestock and aquaculture sectors are heavily impacted, as increased disease prevalence due to climate stressors leads to higher antibiotic usage and reduced productivity [72,98,99,100]. This cycle creates a feedback loop, where more antibiotics are used in response to climate-related health challenges, further driving the increase in resistance as a consequence.

### 3.3. Strategies for Mitigating Economic Impact of Antimicrobial Resistance

In the context of planetary health, the economic implications of AMR are vast and complex, influencing not only healthcare systems but also agriculture and global trade. Moreover, addressing the economic costs of AMR requires a holistic approach that integrates climate change mitigation strategies with efforts to reduce AMU. There is a need to develop an evaluation framework to identify intervention priorities to reduce AMU across clinical, agricultural and environmental settings [101]. More comprehensive economic analyses that account for the long-term costs of climate change on health systems and agricultural productivity will provide a clearer understanding of the financial burden of AMR and help guide policymakers toward effective solutions. Investment in sustainable agricultural practices, improved water and sanitation infrastructure, and global health initiatives aimed at monitoring and controlling AMR are essential [102,103,104]. Ultimately, tackling AMR within the framework of planetary health requires global collaboration, recognizing that the health of humans, animals, and the environment are inextricably linked [90,105,106,107]. As climate change continues to reshape ecosystems and health risks, proactive measures are needed to reduce the spread of AMR, safeguard global health, and mitigate the economic impacts associated with it. Strategic research targeting immunity, complex natural products, and microbiome balance could open new avenues for combatting AMR, with traditional medicine serving as a rich source of complex natural compounds and nature-inspired insights for potent antimicrobial solutions [108]. In addition, strategies focused on investment in research and development and enhancing the healthcare infrastructure, particularly in low- and middle-income countries, can improve the management of resistant infections and reduce economic burdens. In addition, implementing strict regulations on AMU in both human and veterinary medicine can help curb the development of resistance as a part of AMS programs [35,109]. Furthermore, sharing data, resources, and best practices can enhance collective efforts to combat resistance and reduce its economic impact fostering global collaboration [110,111,112]. By investing in research, strengthening healthcare systems, regulating AMU, and fostering global collaboration, we can mitigate the economic burden of AMR and protect the health of our planet.

## 4. Environmental Antimicrobial Resistance Within the Framework of Planetary Health

The environmental dimension of AMR is often overlooked, yet it plays a pivotal role in the spread and persistence of resistant pathogens [113]. In this section, the environmental aspects of AMR are examined within the planetary health framework, highlighting the intricate connections between human activities, environmental health, and the emergence of resistant bacteria since antimicrobial pollution in the environment has a significant impact on the life activities of animals and plants [114]. Furthermore, AMR exemplifies this interconnectedness, as AMU in one domain can influence resistance patterns in another [115,116], while understanding the fate and transport of antibiotics and antibiotic-resistant genes (ARGs) in the environment is crucial for assessing their potential impacts and developing effective mitigation strategies.

### 4.1. Sources of Environmental Antimicrobial Resistance

The environment acts as a reservoir and conduit for antimicrobial-resistant bacteria and genes [117,118]. Actually, the ecosystems serve as a bridge for various compartments of animals to soil to water to sand, and to sewage [119]. Hence, the one health approach is needed because bacteria and bacterial genes often have the ability to move between all three compartments, in any direction [113].

Environmental AMR can be attributed to several key sources, such as pharmaceutical manufacturing, human waste, and the extensive AMU in agriculture and aquaculture [120,121,122,123]. Wastewater treatment plant effluents have been recognized as significant environmental AMR reservoirs due to selective pressure generated by antibiotics that are frequently discharged in water systems [124,125,126]. Actually, several studies have demonstrated that wastewater serves as a hotspot for both antimicrobials and clinically significant antimicrobial-resistant bacteria, as well as resistance genes, suggesting a possible connection between them [127,128,129,130].

The accumulation of antimicrobials in the environment is largely driven by discharges from pharmaceutical manufacturing sites, hospitals, and medical centers, all of which release high concentrations of antimicrobials and their metabolites into wastewater [131,132]. Additionally, antibiotic residues can enter the environment through pharmaceutical manufacturing, improper disposal, agricultural runoff, and the excretion of antibiotics by individuals being treated for infections. When individuals take antibiotics, a portion of the drugs is excreted in their urine or feces, contributing to environmental contamination. These residues can promote the development of resistant bacteria in soil and water, which may then enter the human and animal food chains [132,133].

The extensive AMU in the agriculture, particularly in livestock farming, leads to the excretion of these drugs and their metabolites [134,135]. These substances can enter water bodies and soil through runoff, contributing to the environmental contamination [134,136]. Furthermore, AMU in aquaculture to prevent disease in fish and other aquatic organisms can lead to the release of these substances into surrounding water bodies, fostering resistant strains [131,132]. Moreover, increasing abundances of AMR genes correlate with increased AMU in agricultural settings since it was demonstrated that soil bacterial community structures were consistently altered by the 3-year additions of manure from cattle administered antibiotics compared to soil amended with antibiotic-free manure [133]. The situation is aggravated by the uncontrolled AMU in non-clinical fields (e.g., agriculture, aquaculture, and intensive farming), which accounts for a volume four-times higher than that observed in humans [137].

### 4.2. Environmental Impact of Antimicrobial Resistance Within the Framework of Planetary Health

The presence of antibiotics and resistant bacteria in the environment has several profound effects, such as resistance gene dissemination, an impact on ecosystems, and human and animal health risks. Environmental bacteria can acquire and exchange resistance genes through horizontal gene transfer [138,139]. This genetic exchange can occur in various environmental niches, including soil, water, and sediments, facilitating the spread of resistance [140]. Furthermore, among the effects of antibiotics on ecological functions can be changes in nitrogen transformation, methanogenesis, sulfate reduction, nutrient cycling, and organic matter degradation [141]. Actually, the impact on ecosystem due to antibiotic pollution can disrupt natural microbial communities, which play essential roles in nutrient cycling, organic matter decomposition, and overall ecosystem health and the alteration of these communities can have cascading effects on ecosystem functions [142,143]. In addition, environmental AMR poses a risk to human and animal health since the environmental reservoirs of resistant bacteria can act as sources of infection for humans and animals [119]. Contact with contaminated water, soil, or food can lead to the transmission of resistant pathogens, complicating treatment and control measures [144,145].

### 4.3. Fate and Transport of Antibiotics and Antibiotic-Resistant Genes

The natural environment plays a critical role in developing and spreading AMR by being a reservoir of ARGs [146,147]. Upon entering environmental matrices, antibiotics and ARGs undergo complex processes including adsorption, degradation, transformation, and dilution [143,148]. This mobility underscores the interconnected nature of ecosystems and the challenges in containing and managing these stressors. Once released into the environment, the behavior and persistence of antibiotics are influenced by their physicochemical properties (e.g., solubility, volatility, and sorption capacity), as well as soil characteristics (pH, organic matter, ionic strength, and cation exchange capacity) and weather conditions [142,149,150]. Consequently, the persistence of antibiotics in the terrestrial environment can vary, ranging from less than a day to weeks or even months, primarily depending on factors such as temperature, pH, and the chemical structure of the antibiotic [151].

Human activity exposes soils to pollutants like antibiotics, which promote the spread of AMR. Subinhibitory concentrations of antibiotics increase mutation, recombination, and gene transfer among microbes, allowing resistant strains to rapidly spread. Mobile genetic elements facilitate lateral gene transfer, spreading ARGs alongside genes for resistance to other pollutants like heavy metals and disinfectants [152,153].

Studies have shown that the intramuscular administration of ceftiofur or florfenicol in healthy calves can establish reservoirs of drug-resistant *Escherichia coli* in feces, soil, and bedding [154]. Moreover, the spread of ARGs through contaminated bedding and soil from cows and calves emphasizes the need for strict biosecurity measures [155,156]. Separating treated animals from the herd, avoiding reuse of bedding from infected animals, and cautious use of intramuscular treatments can help mitigate the risk of AMR development. Furthermore, the absorption of antibiotics by crops and aquatic organisms, followed by their integration into the human food chain, raises significant concerns regarding potential public health implications [157,158].

### 4.4. Addressing Environmental Antimicrobial Resistance

Tackling environmental AMR requires a comprehensive approach that integrates efforts across human, animal, and environmental health sectors such as regulation of AMU in all mentioned sectors, enhanced wastewater treatment, as well as surveillance and monitoring of antimicrobial residues and resistant bacteria [63,144,159]. By addressing the fate and transport of antibiotics and ARGs, this section provides a comprehensive overview of their environmental behavior, highlighting the urgent need for integrated, transdisciplinary solutions to mitigate their adverse effects. Furthermore, implementing stringent regulations on the use and disposal of antibiotics in agriculture, aquaculture, and human medicine is crucial [159,160]. In addition, this includes monitoring and controlling pharmaceutical manufacturing discharges [29].

Upgrading wastewater treatment facilities to effectively remove antibiotics and resistant bacteria is essential since advanced treatment technologies, such as membrane filtration and advanced oxidation processes, can significantly reduce environmental contamination [161,162,163,164]. Furthermore, establishing robust surveillance systems to monitor antibiotic residues and resistant bacteria in the environment is vital [165,166]. Finally, it must be recognized that AMR does not respect regional or international borders, making global monitoring crucial for understanding how local policies, practices, and socioeconomic factors impact resistance. Hence, international collaboration and data sharing are essential for establishing baselines and mitigation strategies to preserve antibiotics as a vital resource for future generations [166].

## 5. Social Aspects of Antimicrobial Resistance Within the Framework of Planetary Health

Addressing the social aspects of AMR within the framework of planetary health involves also understanding the interconnectedness of human, animal, and environmental health, as well as the societal behaviors and policies that influence AMR. There are some key social aspects to consider, such as public awareness and education, policy and governance, access to healthcare, agricultural practices and food security, as well as the environmental impact and pollution.

### 5.1. Key Social Aspects of Antimicrobial Resistance

Many people lack awareness of AMR and the role of antibiotic misuse in contributing to resistance. The burden of AMR is expected to be significantly higher in low- and middle-income countries compared to high-income countries, due to several factors, including weak healthcare systems, limited access to quality healthcare, and inadequate diagnostic services [167,168]. In low- and middle-income countries, antibiotics are frequently prescribed for minor illnesses such as viral upper respiratory tract infections or uncomplicated diarrhea, conditions where antibiotics are often unnecessary. Furthermore, in many developing countries, antibiotics are readily available over the counter without medical advice or prescription, contributing to their irrational use and the acceleration of resistance [169]. AMU in livestock, such as poultry, pigs, and cows, raised commercially for food, is widely believed to contribute to AMR [170,171]. Opinions vary on the extent of this impact. Some argue the problem is minimal and well-managed by current public programs, while others highlight greater risks. They are concerned that AMU in animals, whether for treatment or growth promotion, can lead to drug-resistant bacteria (like *salmonella* and *campylobacter*) that may be transmitted to humans through food [172].

In addition to raising awareness among current and future healthcare professionals on AMR issue [62,80,173,174,175], it is also important to raise awareness among general population [176,177]. Furthermore, a study across 12 countries (Nigeria, South Africa, Barbados, Mexico, India, Indonesia, Russia, Serbia, Egypt, Sudan, China, and Vietnam) conducted in 2015 as part of the implementation of objective 1 of the WHO Global Action Plan ‘to improve the awareness and understanding of AMR through effective communication, education and training’ have shown a lack of awareness about AMR and how misuse of antibiotics can lead to resistance among respondents [178]. The raising of awareness of AMR at the European level has been encouraged by the ECDC in the annual European Antibiotic Awareness Day (EAAD), which takes place each year, since 2008, on 18 November. Since November 2015, this event has been expanded to the global level through the World Antibiotic Awareness Week (WAAW) organized by WHO [179]. Hence, education campaigns can help bridge this gap, encouraging responsible AMU and reducing demand for unnecessary prescriptions [174]. However, education alone is not sufficient, it has to be contextualized and behavioral factors and change must be addressed. Influencing individual and collective behaviors regarding AMU is essential [180]. Moreover, the promotion of prosocial behaviors among citizens requires governments, policy makers, and institutions to be oriented toward society [181].

Public awareness campaigns are vital to combating AMR, but they require substantial resources to engage all stakeholders, and raising awareness alone does not necessarily result in behavior change [182]. In the short- and medium-term, targeting specific interest groups, such as healthcare providers or pharmacists, may offer a more effective approach. By directing limited resources towards these key groups, there is a better chance of achieving meaningful changes in behavior. Broader public awareness initiatives can be introduced in subsequent phases, with tailored strategies and messaging to ensure their effectiveness [183].

Influencing both individual and collective behaviors around antibiotic use is critical in combating antimicrobial resistance (AMR). Social norms such as completing prescribed antibiotics, as well as discouraging self-medication play a key role in shaping these behaviors. The optimal duration of a course of oral antibiotics should be sufficient to substantially reduce the patient’s symptoms and prevent relapse, while minimizing adverse effects and the development of antibiotic resistance [184]. Reducing self-medication with antibiotics, often driven by cultural habits or easy access, helps prevent inappropriate use, such as taking antibiotics for viral infections or incorrect dosing. This minimizes the risk of developing resistant bacteria [185].

So, one important social AMR aspect represents policy and governance. International health policies on AMR are strong and enforceable, promoting responsible AMU and addressing environmental contamination [35], but their implementation is still a challenge. In line with WHO’s Global Action Plan on AMR, agreed at the 2017 World Health Assembly, countries have developed their own national action plans on AMR, but the COVID-19 pandemic slowed down their successful implementation [186,187]. Moreover, high-income countries indicated greater progression with achievement of the objectives while low- and middle-income countries presented the need for human and financial resources [188].

One of the social aspects of AMR is access to healthcare since inequities in this field influence AMR rates, especially in low- and middle-income regions where poor implementation, lack of regulation enforcement, low antibiotic awareness, and inadequate distribution of treatment recommendations make effective therapy challenging to obtain [189].

In addition, the social dimension of AMR is deeply intertwined with agricultural practices [73]. Farmers, veterinarians, and policymakers often face economic pressures and the need to maintain competitive productivity, which can lead to the overuse or misuse of antimicrobials while this social pressure is further exacerbated by gaps in regulatory frameworks, lack of education on sustainable practices, and limited access to alternatives, such as vaccines or biosecurity measures. The challenge is not only scientific but also social, requiring behavioral change at multiple levels, from farm management to national policy [190].

Finally, the last key social aspect of AMR is how societies manage pollution and environmental stewardship. Inadequate waste management systems, particularly in low- and middle-income countries, allow for the uncontrolled release of antibiotics into the environment [191]. Additionally, a lack of stringent regulations on industrial and agricultural emissions exacerbates the issue [192]. Social attitudes towards waste disposal, AMU, and environmental protection must shift towards more sustainable practices particularly in addressing AMR and climate change together in achieving the Sustainable Development Goals [97].

### 5.2. Addressing Social Aspects of Antimicrobial Resistance

Considering these social dimensions within the planetary health framework highlights the need for cross-sectoral collaboration and a holistic understanding of how human actions and policies can impact AMR at the global level. Furthermore, addressing the social aspects of AMR requires a multifaceted approach that involves behavior change, improved healthcare infrastructure, stronger regulations, and greater public awareness. Actually, public health campaigns, policy reforms, and increased global cooperation are essential for ensuring that environmental protections are integrated into AMR mitigation strategies [193]. Moreover, the planetary health approach is essential for addressing AMR. By working together across sectors, societies can develop integrated policies that promote sustainable practices, reduce the misuse of antimicrobials, and minimize the spread of resistance.

## 6. Materials and Methods

In this manuscript, we explore the connection between AMU and AMR as an example to illustrate the complex, cross-cutting issues of AMR within the context of human and veterinary medicine, as well as ecosystem, all under the framework of planetary health.

To summarize the work completed to date, we provided an overview of current knowledge on these global challenges and their interconnections. Our literature search was conducted in the PubMed and Web of Science databases, covering articles published from inception to 1 October 2024, using the following search terms: “Planetary health” AND (“Antimicrobial resistance” OR “antimicrobial use”). In total, 77 articles were identified in PubMed, and 50 articles were found in Web of Science.

After removing duplicates, we identified a total of 91 relevant articles, supplemented by additional studies from the reference lists of these articles. Given the broad objective of this overview, we did not apply specific inclusion or exclusion criteria related to publication year, study design, setting, study focus, or particular aspects of the AMR–planetary health relationship. Instead, we included studies that discussed the potential links between planetary health and antimicrobial resistance through the lens of the four pillars of planetary health—social, economic, environmental, and health knowledge—as defined by the Rockefeller Foundation.

## 7. Future Recommendations, Perspectives, Potential Solutions and Feasibility

Combating AMR under the planetary health framework requires interdisciplinary strategies targeting social, economic, environmental, and health dimensions. Key recommendations include enhancing public education on responsible AMU, implementing robust stewardship programs across sectors, improving pharmaceutical waste management, and monitoring environmental dissemination of antimicrobials (Table 1). Potential solutions include rapid diagnostic tools, advanced wastewater treatment technologies, and the development of antimicrobial alternatives. Sustainable agricultural practices, such as precision farming, can also reduce AMU. Feasibility depends on global collaboration, investment in innovative technologies, and the integration of these strategies into policy frameworks to ensure long-term effectiveness.

Collaborative research, data sharing, and policy integration are critical for addressing AMR globally, alongside promoting innovative alternatives to antibiotics and sustainable agricultural practices. These efforts underscore the necessity of a holistic, synergistic approach to safeguard ecosystems, public health, and sustainable development.

## 8. Conclusions

In this research, the importance is underscored of adopting a planetary health perspective to address the systemic challenges posed by AMR. This collaboration must extend beyond traditional healthcare boundaries, encompassing policy development, public education, and environmental management to address AMR comprehensively. The planetary health approach adds a critical dimension to the one health framework by recognizing the broader environmental, social, and economic factors influencing the development and spread of AMR. It emphasizes the importance of ecological sustainability, climate action, and environmental stewardship as essential components in reducing AMR. Key findings highlight the interconnectedness of AMR with ecological sustainability, climate action, and environmental stewardship, which are essential components in mitigating AMR. By integrating these factors, the need for a more coordinated and comprehensive response to AMR is emphasized in this research. This perspective encourages a holistic, transdisciplinary approach, uniting experts from diverse sectors such as healthcare, environmental sciences, policy, and education. Ultimately, a synergistic approach that unites human and veterinary medicine, along with a collective global effort, is vital for reducing the global AMR burden, ensuring sustainable practices, and safeguarding the efficacy of antibiotics for future generations.

## Figures and Tables

**Table 1:** Key recommendations to combat AMR under the planetary health framework.

**Section**	**Recommendations**
Social Awareness	Enhance public awareness and education on AMR through targeted campaigns and community engagement
Healthcare Practices	Implement antimicrobial stewardship programs in both human and veterinary medicine to promote the prudent use of antibiotics
Policy Development	Develop and enforce policies that regulate antimicrobial use in agriculture, healthcare, and environmental management
Environmental Management	Reduce pharmaceutical waste, agricultural runoff, and industrial pollution to limit the spread of resistant pathogens
Research and Innovation	Support interdisciplinary research focusing on the environmental, social, and economic aspects of AMR
Global Collaboration	Foster global collaboration between sectors, nations, and organizations to create comprehensive AMR management strategies

## Data Availability

Not applicable.
